# Machine learning-based analysis of regional differences in out-of-hospital cardiopulmonary arrest outcomes and resuscitation interventions in Japan

**DOI:** 10.1038/s41598-023-43210-x

**Published:** 2023-09-23

**Authors:** Yasuyuki Kawai, Koji Yamamoto, Keita Miyazaki, Hideki Asai, Hidetada Fukushima

**Affiliations:** Department of Emergency and Critical Care Medicine, Nara Medical University, 840 Shijo-Cho, Kashihara, Nara 634-8522 Japan

**Keywords:** Therapeutics, Medical research

## Abstract

Refining out-of-hospital cardiopulmonary arrest (OHCA) resuscitation protocols for local emergency practices is vital. The lack of comprehensive evaluation methods for individualized protocols impedes targeted improvements. Thus, we employed machine learning to assess emergency medical service (EMS) records for examining regional disparities in time reduction strategies. In this retrospective study, we examined Japanese EMS records and neurological outcomes from 2015 to 2020 using nationwide data. We included patients aged ≥ 18 years with cardiogenic OHCA and visualized EMS activity time variations across prefectures. A five-layer neural network generated a neurological outcome predictive model that was trained on 80% of the data and tested on the remaining 20%. We evaluated interventions associated with changes in prognosis by simulating these changes after adjusting for time factors, including EMS contact to hospital arrival and initial defibrillation or drug administration. The study encompassed 460,540 patients, with the model’s area under the curve and accuracy being 0.96 and 0.95, respectively. Reducing transport time and defibrillation improved outcomes universally, while combining transport time and drug administration showed varied efficacy. In conclusion, the association of emergency activity time with neurological outcomes varied across Japanese prefectures, suggesting the need to set targets for reducing activity time in localized emergency protocols.

## Introduction

Out-of-hospital cardiac arrest (OHCA) is a global health problem with poor outcomes^1,2^. Although international resuscitation guidelines exist^3,4^, countries and regions adapt them to their local emergency medical services (EMSs)^5–8^, resulting in fragmented protocols and challenges in identifying improvement measures across regions. It remains unclear whether interventions associated with improved outcomes in one region will be effective in another.

Therefore, this study aimed to use machine learning to analyze emergency activity records from 47 Japanese prefectures to identify regional differences in time reduction strategies associated with improved outcomes. We hypothesized that targets for reducing EMS activity time would vary regionally owing to different adapted protocols.

We previously reported the potential of machine learning in predicting neurological outcomes from EMS activity records in a region that followed a single protocol^9^ but did not consider its generalizability in other regions. Our study extends this research by analyzing records across multiple Japanese regions with different protocols.

We developed a machine learning model to predict neurological outcomes using the 47 prefectures as predictors in the Utstein-style EMS records. Subsequently, we visualized and compared the association of increasing or decreasing EMS activity time with outcomes for each prefecture.

## Methods

### Study design

We conducted a retrospective study utilizing prospectively recorded Japanese Utstein-style EMS activity records. The Ethics Committee of Nara Medical University approved the study (No. 3353), and the requirement for informed consent was waived owing to the use of anonymized records. This study was conducted in accordance with the tenets of the Declaration of Helsinki.

### Study population and data collection

Japan has an aging population as 28.9% of its 130 million people are aged > 65 years^10^. The country consists of 47 prefectures with varying population densities of 65.4–6,399.5 individuals/km^2^. EMSs respond to all emergency calls and transport approximately 125,000 patients with OHCA to hospitals annually^11^. Emergency protocols, based on the Japanese Resuscitation Council’s Resuscitation Guidelines^12^ and revised every 5 years, are developed and implemented by 250 regional health managers. Each medical control region is supervised by a council established in each prefecture, tailoring protocols to local conditions^13–15^. EMS activities are recorded in the Utstein style and verified by the medical control council, and all records are collected annually by the Fire and Disaster Management Agency^11^. Our analysis included prehospital records of patients with OHCA resuscitated by EMS and transported to hospitals in 47 prefectures between 2015 and 2020, excluding patients aged < 18 years and those with non-cardiogenic cardiopulmonary arrest to reduce pathology variability.

### Investigating Japanese EMS practices

In Japan, EMS is activated via a Communications Command Center upon receiving emergency calls. Bystanders may be instructed to administer cardiopulmonary resuscitation (CPR) over the telephone if cardiac arrest is suspected. Each ambulance includes a team of three, often featuring emergency life-saving technicians capable of advanced airway management and adrenaline administration for OHCA, under online medical control supervision. Additionally, hospital destinations are determined during field operations, and all patients, barring those with evident signs of death, are transported to a hospital.

### Data collection and pre-processing

We employed 23 factors and prefecture numbers from the Utstein-style EMS activity records as predictors, including county number, age, year and month of onset, bystander type, initial rhythm, number of defibrillations, number of adrenaline boluses administered, and elapsed time of each activity. Notably, the prefecture number was treated as a continuous variable due to its sequential allocation from north to south. This approach aimed to capture potential spatial correlations between adjacent prefectures. We also conducted a similar analysis using one-hot encoding for the prefecture numbers, and the outcomes did not contradict the results obtained when treating the prefecture number as a continuous variable. Categorical data were one-hot encoded. Remarkably, in the case of missing data, we refrained from substituting them with any particular value. Instead, the data missingness was coded as a separate category, which was incorporated into our analysis as a separate data element. Selected continuous variables were standardized using z-score normalization, a method that confers advantages in machine learning algorithms such as neural networks by aiding gradient descent convergence and mitigating issues related to weight initialization and gradient problems. Time factors, which were initially considered continuous variables, were one-hot encoded as categorical data^16^ because of their non-linear relationship with prognosis in cardiopulmonary resuscitation. The time factors were measured in minutes and thus represented as 1, 2, 3, 4, … minutes.

Cases in which a specific intervention, such as defibrillation or drug administration, was not performed were also considered. These were coded as “no intervention” and incorporated into the contact-to-intervention column, allowing the model to reflect a comprehensive range of patient experiences. These steps resulted in 249 features (see Supplementary Table S1). Subsequently, we constructed a machine learning model to predict good neurological outcomes 1 month after cardiac arrest, based on the cerebral performance category (CPC) score^17^—a binary classification (Yes/No), with CPC1/2 signifying good neurological outcome and CPC3-5 indicating poor neurological outcome—sourced from the Utstein records.

### Dataset selection and predictive model development

We stratified and randomly split the training and test datasets using an 8:2 ratio based on CPC1/2 to ensure a consistent ratio for predictive model construction. The prediction model was built using the neural network with the best average class sensitivity after several machine learning model trials. The compared methods included logistic regression, support vector machine, decision tree, random forest, and LightGBM^9^. To balance model bias (underfitting) and variance (overfitting), we applied a stratified cross-validation method (five-fold) using CPC1/2, along with batch normalization and dropouts in each neural network layer. The model’s accuracy plateaued after increasing the number of layers to five because of which we used a five-layer network to optimize learning costs. The sigmoid function served as the activation function and binary cross-entropy served as the loss function^18^. We measured model performance using area under the receiver operating characteristic curve (AUROC) and accuracy during training.

Imbalanced datasets significantly affect minority class performance. To address misclassification, we simulated based on predicted CPC1/2 numbers and employed class weighting during training to balance sensitivities, considering trade-offs. Our model aimed to maximize the majority class (CPC3–5) sensitivity without excessively reducing minority class (CPC1/2) sensitivity. We set CPC1/2 sensitivity at 80% and tested weights from 1 to 100 in 0.1 increments to optimize CPC3-5 sensitivity.

Additional training parameters included a batch size of 1,024,100 epochs, a learning rate of 0.001, and Adam optimizer. We conducted training using Python version 3.8.5 (Python Software Foundation, Beaverton, OR, USA).

### Adjusting time parameters in the simulation method

We assessed the association of EMS activity duration with predicted CPC1/2 counts by simulating the constructed prediction model on a test dataset (n = 92,108), containing all previously split prefectures from the training set. The simulation methodology involved three time factors: elapsed time from EMS arrival to hospital arrival (a), EMS arrival to first defibrillation (b), and EMS arrival to first drug administration (c).

Previous studies have shown that these temporal factors are important prognostic predictors of EMS activity time^19–26^. For example, shorter time from EMS arrival to defibrillation^19,25^ and from EMS arrival to drug administration^20–25^ are associated with better survival and improved neurological outcomes in OHCA patients. The prognostic impact of EMS providers staying on scene and performing their activities has also been reported^26^. Patients with non-shockable initial rhythm were excluded for (b), and those with EMS-witnessed cardiac arrest were excluded for (c). Time factors increased or decreased by − 5 to + 5 min for defibrillation and drug administration, and from − 5 to + 10 min for EMS arrival to hospital arrival time, in 1-min increments. We created a dataset adjusting each time factor in the test dataset and calculated the average predicted CPC1/2 score using the created prediction model. Then, we determined the percentage change in mean predicted CPC1/2 count to assess the association of time increase/decrease with the unadjusted data. We focused on percentage change relative to unadjusted data for a prefecture-specific analysis. A heat map visualized and evaluated the proportion of change between time adjustment and mean predicted CPC1/2 count.

### Comparison of predicted changes of CPC1/2 counts across prefectures

We employed the same time adjustment method to estimate and visualize predicted CPC1/2 counts for the test dataset split by prefecture. We identified the time adjustments most associated with prognosis in each prefecture for the combinations (a) & (b) and (a) & (c), revealing treatment and EMS arrival to hospital arrival time adjustments with the greatest potential to improve predicted prognosis.

### Statistical Analyses

Patient characteristics are summarized as medians and interquartile ranges (IQRs) for continuous variables and counts and percentages for categorical variables. Additionally, the evaluation metric for the five models is expressed as means ± standard deviations. The standard deviations were calculated based on the variations in the evaluation metric across the five-fold cross-validation.

## Results

We analyzed data from 753,910 patients with OHCA who received CPR by EMS during the study period. After applying the inclusion criteria (Supplementary Figure S1), 460,540 (61%) cases were included. Table 1 summarizes patient characteristics, with a mean age of 81 (IQR: 70–88) years and 57% male individuals. Missing data were identified and newly coded for witness type information (7.2%), bystander chest compressions (21.5%), bystander ventilation (38.3%), and airway securement (0.002%). For the three time intervals, the adjusted percentages of patients were 100%, 9.2%, and 95.6% for EMS to hospital arrival, first defibrillation, and first drug administration, respectively.

**Table 1:** Patient background characteristics.

Variables	Group	Overall
		n = 460,540
Age, years		81 [70, 88]
Male sex, n		264,009 (57.3)
Onset year, n	2015	72,949 (15.8)
	2016	74,329 (16.1)
	2017	77,587 (16.8)
	2018	78,738 (17.1)
	2019	78,189 (17.0)
	2020	78,748 (17.1)
Witness, n		186,032 (40.4)
Type of witness, n	Family	92,027 (20.0)
	Friend	6,700 (1.5)
	Colleagues	6,139 (1.3)
	Passengers	6,405 (1.4)
	Others	40,670 (8.8)
	Firefighter	823 (0.2)
	Paramedic	14,987 (3.3)
	Emergency lifesaver	19,770 (4.3)
	Unknown	33,195 (7.2)
Bystander, n		235,513 (51.1)
Bystander chest compression, n		231,842 (50.3)
	Unknown	98,787 (21.5)
Bystander rescue breathing, n		28,213 (6.1)
	Unknown	176,558 (38.3)
Bystander defibrillation, n		9,891 (2.1)
EMS with emergency lifesaver, n		455,658 (99)
EMS with medical doctor, n		14,712 (3)
Initial rhythm, n	VF	41,115 (9)
	Pulseless VT	1,295 (0.3)
	PEA	97,913 (21)
	Asystole	300,063 (65)
	Other	20,154 (4)
Defibrillation, n		60,068 (13)
Time from contact to first defibrillation, min		2 [1, 8]
Defibrillation frequency, times		0 [0, 0]
Medication administered, n		107,962 (23)
Time from contact to first administration, min		14 [10, 19]
Administration frequency, times		0 [0, 1]
Airway management with instruments, n	Yes	397,646 (86)
	Unknown	2,321 (0.5)
Time from call to contact, min		9 [7, 11]
Time from contact to arrival, min		23 [18, 30]
ROSC, n		47,026 (10)
CPC ½, n		20,618 (5)

*IQR* interquartile range; *EMS* emergency medical service; *VF* ventricular fibrillation; *VT* ventricular tachycardia; *PEA* pulseless electrical activity; *ROSC* return of spontaneous circulation; *CPC* cerebral performance category.

Continuous variables are presented as median (IQR). Categorical variables are presented as n (%).

**Figure 1:**
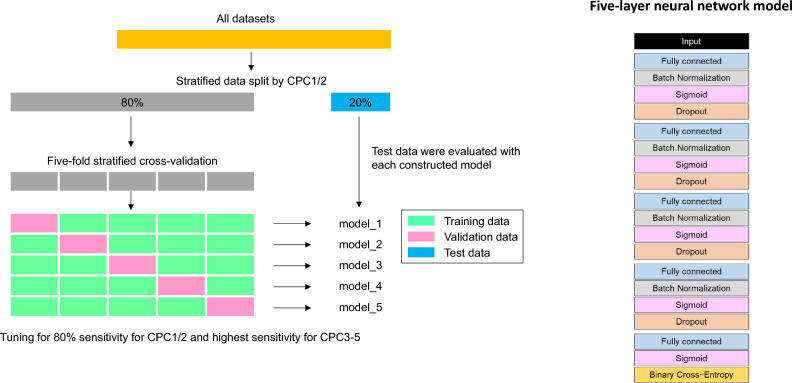
Overview of data splitting and stratified cross-validation methods and the neural network-based machine learning model. The model was developed using the stratified cross-validation method with CPC1/2. The machine learning model consisted of a five-layer neural network. AUROC—area under the receiver operating characteristic; BN—batch normalization; CPC—cerebral performance category.

Our predictive models (Fig. 1) were established based on the abovementioned features and showed remarkable accuracy and sensitivity in predicting patient outcomes. Specifically, the AUROC curve and accuracy for the validation and test data were 0.96 ± 0.00 and 0.96 ± 0.00 as well as 0.96 ± 0.00 and 0.95 ± 0.00, respectively. Sensitivity of CPC1/2 and CPC3-5 for test data, including all prefectures, was 0.80 ± 0.01 and 0.96 ± 0.00, respectively (Supplementary Figure S2, which further illustrates the model performance across all prefectures). This comprehensive sensitivity analysis supports the robustness of our findings, thereby affirming the validity of our subsequent, more detailed investigations.

**Figure 2:**
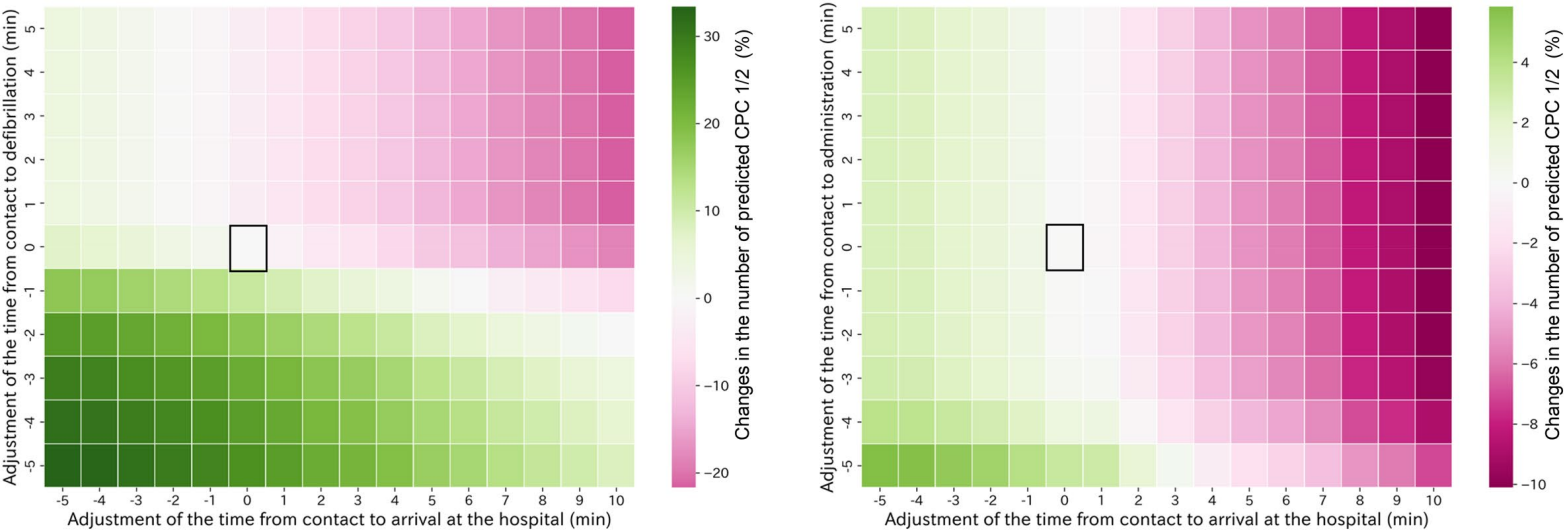
Associations of changes in EMS activity time with predicted CPC1/2 in 47 prefectures. No adjustments are represented by square boxes. The color bar indicates the increase or decrease in predicted CPC1/2 relative to the unadjusted case. The left panel displays adjusted results for EMS arrival to hospital arrival time and to first defibrillation time. The right panel presents adjusted results for EMS arrival to hospital arrival time and EMS arrival to first drug administration time. In both scenarios, shorter activity times improved prognosis, while longer activity times worsened it. However, the changes ranged from − 20 to + 30 and from − 10 to + 5 for each factor. CPC—cerebral performance category; EMS—emergency medical service

When delving into the impact of EMS activity time factors, we gauged their combined prognostic influence on the test data, encompassing all prefectures. This analysis demonstrated compelling patterns, as presented in Fig. 2. Figure 2 (left) shows a heatmap adjusted for the EMS arrival to hospital arrival and first defibrillation times, with decreases and increases in both time factors having an additive relationship with the predicted CPC1/2 count. Similarly, Fig. 2 (right) is adjusted for the EMS arrival to hospital arrival and first drug administration times, with the prognostic association of EMS arrival to hospital arrival time being more substantial than the EMS arrival to drug administration time. However, our findings emphasize that the outcome association with both time factors combined is not just the monotonic influence of a single factor but an additive association of two factors over the time range. Intriguingly, we observed diverse changes ranging from -20% to + 30% in predicted CPC1/2 counts adjusted for the EMS arrival to hospital arrival time and EMS arrival to first defibrillation time. This range was larger than the changes in predicted CPC1/2 counts adjusted for the EMS arrival to hospital arrival time and EMS arrival to first drug administration time, which was − 10 to + 5%.

**Figure 3:**
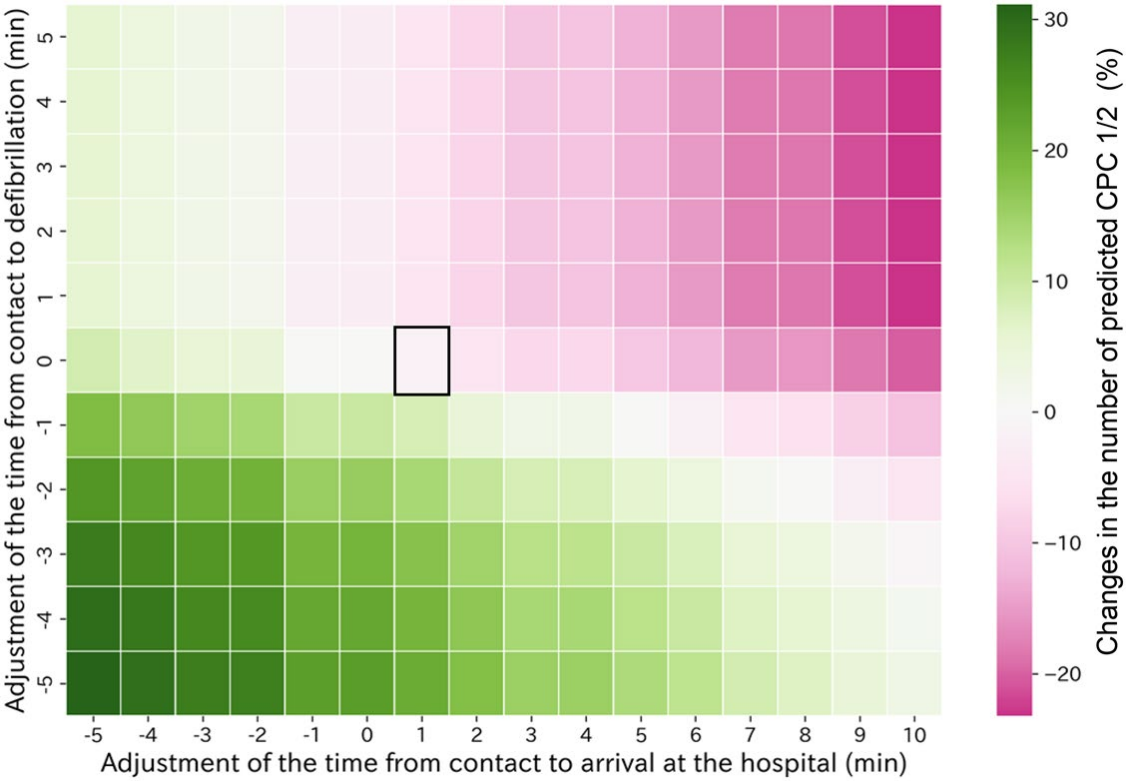
Example of the association of changes in EMS arrival to hospital arrival time and defibrillation time with predicted CPC1/2. No adjustments are represented by square boxes. The color bar indicates the increase or decrease in predicted CPC1/2 relative to the unadjusted case. The figure demonstrates a consistent observation across all 47 prefectures that a decrease in the time intervals between EMS arrival to hospital arrival time and to first defibrillation time is anticipated to enhance patient prognosis. The observed changes spanned from − 20 to + 30 and − 10 to + 5. EMS—emergency medical service; CPC—cerebral performance category.

**Figure 4:**
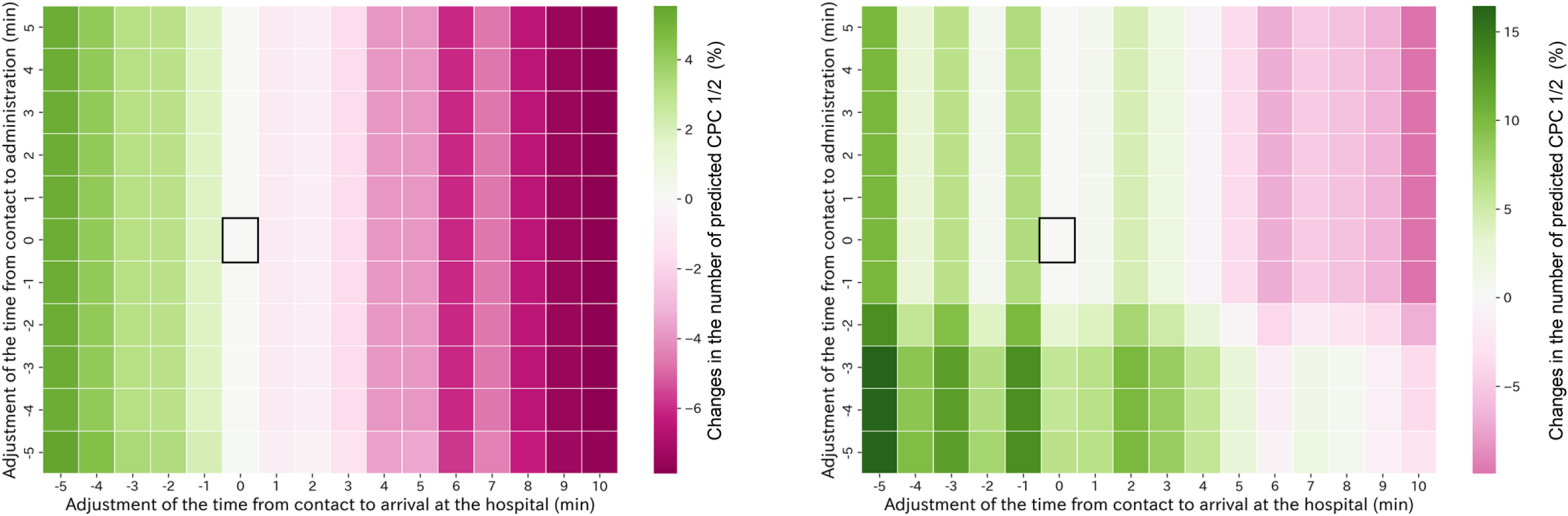
Example of the association of changes in EMS arrival to hospital arrival time and administration time with predicted CPC1/2. No adjustments are represented by square boxes. The color bar indicates the increase or decrease in predicted CPC1/2 relative to the unadjusted case. In the prefecture shown in the left panel, shortened EMS arrival to hospital arrival time was associated with improved prognosis; no association was seen with EMS arrival to drug administration time. However, in the prefecture shown on the right, earlier drug administration improved prognosis more than shorter EMS arrival to hospital arrival time. In contrast to defibrillation, different associations were observed in different prefectures. Changes ranged from − 7 to + 5 and − 10 to + 15 for each factor. EMS—emergency medical service; CPC—cerebral performance category.

The Figs. 3 and 4 display simulation results for representative prefectures, while Supplementary Figures S3 and S4 provide an animated sequence of results for all prefectures. Reducing the time to first defibrillation consistently increased the predicted CPC1/2 count across all prefectures, whereas longer EMS arrival to hospital arrival time had the opposite association (Fig. 3). However, the association of drug administration and EMS arrival to hospital arrival time with patient outcomes varied among prefectures. For example, in the prefecture shown in Fig. 4 (left), changes in drug administration time did not influence the predicted CPC1/2 count, but a decrease in EMS arrival to hospital arrival time increased it. In contrast, in the prefecture shown in Fig. 4 (right), earlier drug administration improved prognosis more than shorter EMS arrival to hospital arrival time. These variances underscore the importance of understanding the local context when interpreting the associations of these factors with predicted outcomes.

## Discussion

In this study, we examined Japanese EMS records and neurological outcomes from 2015 to 2020 using nationwide data. The study provided valuable insights into the association between EMS activity time and predicted neurological outcomes of patients with OHCA using a machine learning model that accounts for regional variations in emergency medical protocols. Interestingly, the findings suggested that the optimal interventions to improve EMS performance may differ depending on a region’s medical background and EMS protocols. This highlighted the importance of tailoring interventions to the specific needs of each region rather than using a one-size-fits-all approach.

Prediction of neurological outcome after cardiac arrest by machine learning reportedly improves accuracy compared with traditional methods^27–30^. The novelty of this study lies in our independent adjustment of the balance between the majority and minority groups, which was essential because our objective was focused on the number of predictions for a good neurological prognosis. However, even after this adjustment, we obtained AUROCs comparable to those of previous studies. This finding underscores the robustness and reliability of our methodology. Developing models with high predictive accuracy and simulating the association of multiple intervention factors is a promising approach for assessing the prognostic association of different combinations of interventions. Previous studies to improve resuscitation have only accepted interventions with positive associations, based on evidence from statistical methods^31–33^. Simulation by machine learning models can theoretically change any parameter within the range of the training data^30,34,35^. Simulation can also be done at any time, as long as the data set is available, and is less susceptible to social changes, such as those arising from coronavirus pandemics. In this study, conducting and comparing this simulation on a county-by-county basis, which were considered to have different backgrounds, led us to conclude that the time-saving factors that are expected to improve prognosis the most, differ from county to county.

However, as shown in a previous study^9^, the range of possible simulations is limited by the diversity of the data set because of which a large data set must be collected to increase the diversity. The Utstein style is widely used worldwide, and therefore, seems to be suitable for building other specific and general models using data from different backgrounds^36^. In Japan, especially, all patients receiving emergency services treatment are recorded using the Utstein style, enabling comprehensive data collection^37^. By recoding missing values as machine learning features, the risk of selection bias due to missing values is mitigated. In this study, only 0.3% of cases were excluded owing to missing or negative time series data or activity time longer than 24 h (Supplementary Figure S1).

The simulations conducted in this study revealed that the association of EMS arrival to hospital arrival time and medication on outcomes varied among prefectures. These differences may be attributed to variations in EMS protocols, technical proficiency, and geographical conditions, but this is unknown as this study did not aim to identify these factors. However, by identifying the interventions that have the strongest association with outcomes in a particular region, these findings could inform the development of tailored interventions that are most suitably associated with positive outcomes for that region. Furthermore, it would be possible to suggest the time reductions that should be prioritized if the target of the activity is time reduction. Overall, this study underscores the importance of taking a region-specific approach to improve EMS performance and highlights the potential of machine learning models to identify the interventions exhibiting the strongest association with desired outcomes for a given region.

### Limitations

Our study has some limitations that should be addressed in future research. First, the predictors were restricted to data from the Utstein-style EMS activity records, which only provided categorical data on activity absence or presence and continuous data on time. Therefore, the technical quality of EMS activities and interventions at the destination hospitals were not included as predictors, potentially limiting the accuracy of the neurological outcome prediction models. Additionally, geographical factors, such as access to emergency services and hospitals, were not considered. Second, the potential range of simulations was confined to the range of activities performed by EMS, preventing the evaluation of the association of increased or decreased time for unimplemented activities. A diverse training dataset encompassing a wide range of EMS activities is required to address this limitation. Furthermore, the analyzed EMS activity records from 2015 to 2020 may not reflect the latest life-saving practices. In addition, as this study focused on EMS activities in Japan, its findings may not be directly generalizable to other countries. Third, although the study compared the association of EMS activity time at a prefectural level, EMS protocols might have been developed for more subdivided regions. This study was based on the smallest division where information could be collected (i.e., prefectures). More detailed regional comparisons could suggest emergency activity targets for individual protocols tailored to each region, potentially leading to a general model applicable to individual hospitals with unavailable EMS data. Finally, the feasibility of the simulation results should be acknowledged. Although machine learning models can provide valuable insights, their association with desired outcomes in real-world clinical settings may vary due to factors, such as patient characteristics and provider’s expertise. To improve the applicability and clinical utility of these models, future research should focus on validating them in real-world settings and addressing potential barriers to implementation.

## Conclusions

This study highlights the regional differences in EMS activity time targets and their implications in tailored prehospital care. The study findings may help enhance in EMS protocols and improve patient outcomes. However, it is crucial to address the identified limitations to strengthen our recommendations.

### Supplementary Information

Supplementary Information 1.

Supplementary Information 2.

Supplementary Video 1.

Supplementary Video 2.

Supplementary Information 3.

Supplementary Information 4.

## Data Availability

The data that support the findings of this study are available from the corresponding author, Y.K, on reasonable request.
