# Supplementary material for: Machine learning-based analysis of regional differences in out-of-hospital cardiopulmonary arrest outcomes and resuscitation interventions in Japan
Source: Sci Rep. 2023 Sep 23;13:15884. doi: 10.1038/s41598-023-43210-x (PMC10518013; doi:10.1038/s41598-023-43210-x)
Supplement: Supplementary file 2 — Supplementary Information 2. [file 41598_2023_43210_MOESM2_ESM.pptx]

## Slide 1
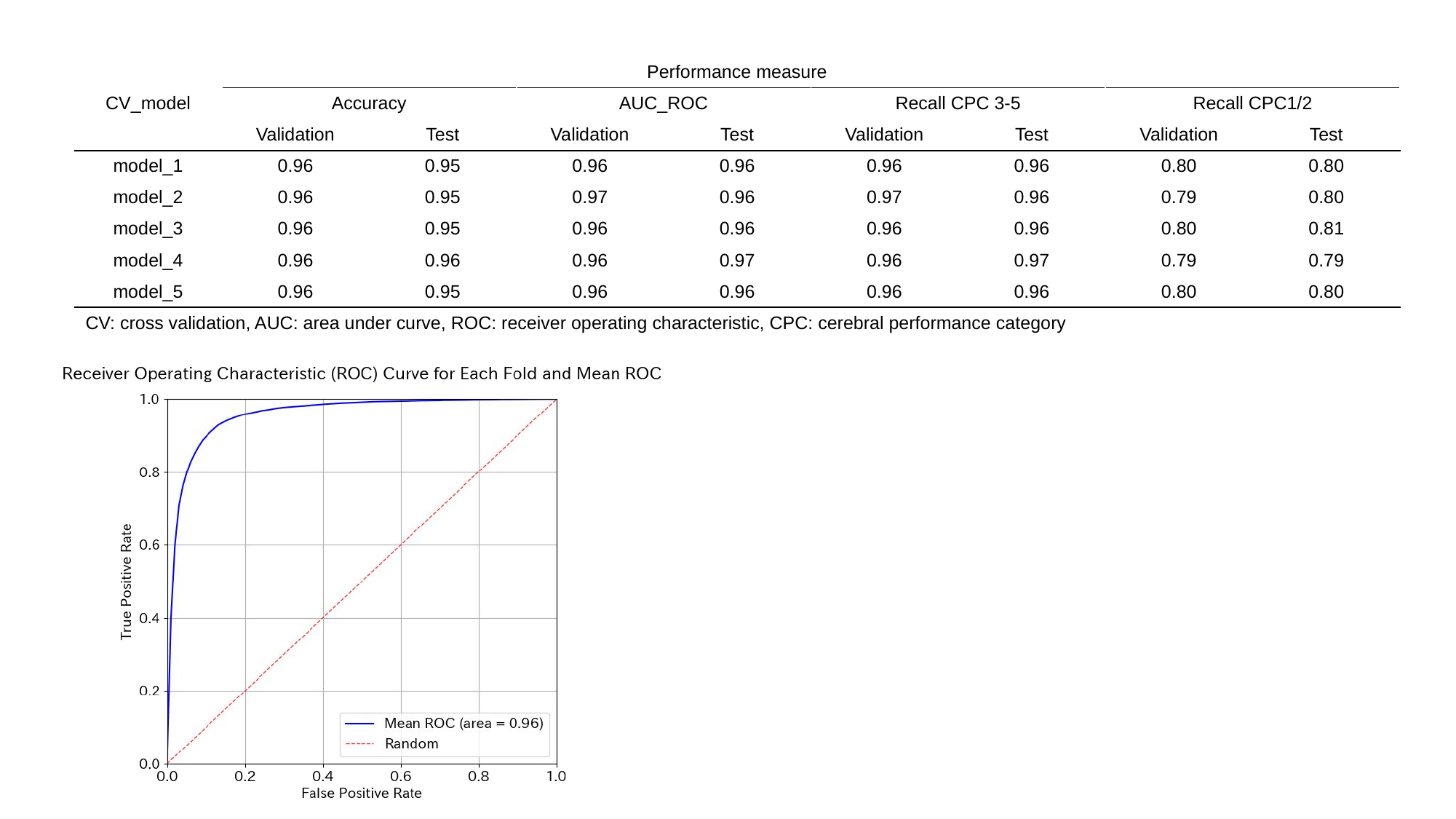

| | Performance measure | | | | | | | |
| --- | --- | --- | --- | --- | --- | --- | --- | --- |
| CV\_model | Accuracy | test\_accuracy | AUC\_ROC | test\_auc\_roc | Recall CPC 3-5 | test\_recall\_CPC3-5 | Recall CPC1/2 | teset\_recall\_CPC1/2 |
| | Validation | Test | Validation | Test | Validation | Test | Validation | Test |
| model\_1 | 0.96 | 0.95 | 0.96 | 0.96 | 0.96 | 0.96 | 0.80 | 0.80 |
| model\_2 | 0.96 | 0.95 | 0.97 | 0.96 | 0.97 | 0.96 | 0.79 | 0.80 |
| model\_3 | 0.96 | 0.95 | 0.96 | 0.96 | 0.96 | 0.96 | 0.80 | 0.81 |
| model\_4 | 0.96 | 0.96 | 0.96 | 0.97 | 0.96 | 0.97 | 0.79 | 0.79 |
| model\_5 | 0.96 | 0.95 | 0.96 | 0.96 | 0.96 | 0.96 | 0.80 | 0.80 |
| CV: cross validation, AUC: area under curve, ROC: receiver operating characteristic, CPC: cerebral performance category | | | | | | | | |
