# Supplementary material for: Machine learning-based analysis of regional differences in out-of-hospital cardiopulmonary arrest outcomes and resuscitation interventions in Japan
Source: Sci Rep. 2023 Sep 23;13:15884. doi: 10.1038/s41598-023-43210-x (PMC10518013; doi:10.1038/s41598-023-43210-x)
Supplement: Supplementary file 6 — Supplementary Information 4. [file 41598_2023_43210_MOESM6_ESM.docx]

**Supplementary Figure Legends**

**Supplementary Fig. S1. Flow chart of patient selection**

**Supplementary Fig. S2. Results of the five valuations using the stratified cross-validation method and receiver operating characteristic (ROC) curve for each fold and mean ROC**

The table above shows performance indicators for each model evaluated using cross-validation, while the figure below shows the mean receiver operating characteristic curves for the five models.

**Supplementary Fig. S3. Animation of combined simulation: all prefectures, arrival and defibrillation times**

Across all prefectures, reductions in transport time and time to first defibrillation consistently enhanced the predicted number of CPC1/2 cases

CPC, cerebral performance category

**Supplementary Fig. S4. Animation of combined simulation: all prefectures, arrival and drug administration times**

Different prefectures show varying contributions from transport time reduction and time to first drug administration in increasing the predicted number of CPC1/2 cases.

CPC, cerebral performance category
